# The Role of Random Texture Scattering on the Absorptance Enhancement in Halide Perovskite Layers

**DOI:** 10.1021/acsami.5c09757

**Published:** 2025-08-20

**Authors:** Meng-Hsueh Kuo, Branislav Dzurňák, Neda Neykova, Lucie Landová, Ivana Beshajová Pelikánová, Zdeněk Remeš, Chih-Yu Chang, Stefaan De Wolf, Jakub Holovský

**Affiliations:** † Centre for Advanced Photovoltaics, Faculty of Electrical Engineering, 48220 Czech Technical University in Prague, Technická 2, 16627 Prague, Czech Republic; ‡ Institute of Physics, Czech Academy of Sciences, Cukrovarnická 10, 16200 Prague, Czech Republic; § Department of Materials Science and Engineering, 34878 National Taiwan University of Science and Technology, 10607 Taipei, Taiwan; ∥ KAUST Solar Center (KSC), 127355 King Abdullah University of Science and Technology (KAUST), 23955-6900 Thuwal, Saudi Arabia

**Keywords:** halide perovskite, Yablonovitch model, Poruba model, nanotexture, substrate roughness, light trapping, light scattering, light absorptance

## Abstract

Hybrid perovskites are a class of thin-film semiconductors with remarkably steep absorption edges and high absorption coefficient. In the case of solar cells, a film thickness of less than a micrometer is usually sufficient to absorb most of the light when combined with a back reflector. Otherwise, an efficient light trapping strategy may be desired, e.g., in the case of tandem or semitransparent cells. Traditionally, light trapping is accomplished by employing randomly nanotextured substrates. In this contribution, absorption enhancements due to not only nanorough but also microrough substrates and with or without additional gold coating are evaluated from the point of gains in photocurrent and from the point of view of valid optical models. We find that light trapping from nanotextured substrates follows mainly the Yablonovitch model, leading to an apparent shift of absorption edge. This contrasts with microrough substrates and also the remarkable efficient light trapping capabilities of bare layers due to their native surface roughness, where the path enhancement in this case is almost uniform, making the layer optically thicker by factor two or more. Light trapping optical models as well as analytical techniques are reviewed, and new insights are presented.

## Graphical abstract

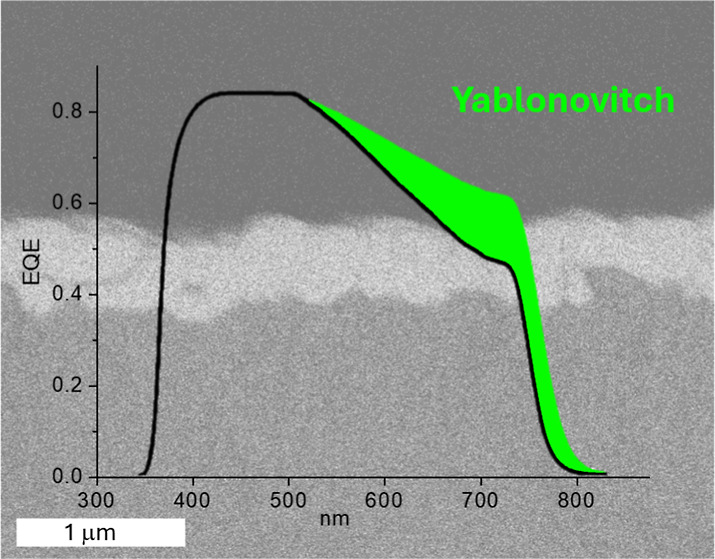

## Introduction

Hybrid organic–inorganic lead halide perovskites are a class of semiconductor thin-film material with remarkable optoelectronic properties, being currently the only wide bandgap top cell candidates for tandem devices working with crystalline silicon bottom cells exceeding 30% efficiency, as first demonstrated by researchers at EPFL/CSEM Neuchâtel,^1^ then further improved by other academic groups,^2^ until the record was finally taken over by industry company Longi currently with 34.85% efficient device.^3^ Especially in tandem cells, where it is not possible to apply a back reflector behind the perovskite layer, incomplete absorptance leads to transmittance of photons through the top cell, leading to overlapping components of the external quantum efficiencies of the top and bottom cells in the range of 600 nm – 800 nm.^4,5^ The front micrometer scale-textured surface considerably reduces this effect due to geometric light path enhancement as can be seen from comparison of tandems with flat and textured front surface from the same laboratory.^1,6^ A similar geometrical optical path enhancement concept was successfully demonstrated by glass texturing.^7^ The use of periodic nanostructuring for light trapping was studied extensively theoretically, e.g., based on pyramidal surface^8,9^ inverted cones^10^ or ZnO photonic crystals.^11^ Experimentally, this was demonstrated, e.g., by diffraction gratings, making use of CD and DVD discs.^12^ Conversely, random texturing of transparent conductive oxide (TCO) substrates was experimentally demonstrated only with moderate success,^13,14^ mainly due to very low contrast of refractive indices^15^ between the TCO and perovskite layers. More successful was therefore concept or random nanotexturing of the back reflector^16^ where the contrast was guaranteed by an interface with metal. Metal interfaces provide plasmonic effects that do increase useful absorptance^17–19^ but principally also the parasitic absorptance can be increased.^20^ The interface between the perovskite and air also provides sufficient refractive index contrast. In early days of perovskite solar cell technology, idealized Yablonovitch limit path enhancement^21^ for such a case was evaluated analytically. The Yablonovitch limit represents a theoretical model^22^ giving maximum path enhancement of 2*n* ^2^ (4*n* ^2^for back reflector) where *n* represents the layer refractive index. (For CH_3_NH_3_PbI_3_ layer we use value *n* = 2.5 here.) This factor comes from intensity enhancement in medium with higher refractive index *n* compared to vacuum according to *I*_int_ = *n* ^2^ *I*_ext_.^22^ As we deal with local enhancement, where the reference intensity is that of the near ambient that may not be air but glass or a liquid, we obviously have to account for its refractive index *n*_amb_ too. The limit can be written as follows^23^

1 $$A_{Yablonovitch}≅{\left[1+\frac{1}{2\alpha {\left(n/n_{amb}\right)}^{2}d}\right]}^{-1}$$

where the refractive index is reduced by the refractive index of ambient *n*_amb_, α is the absorption coefficient, and *d* is the layer thickness. eq 1 was derived only for the case where α*d* ≪ 1, however, for the purposes of approximate determination of solar cell efficiency limits, it is sometimes used in the whole range. Another model, based on scalar scattering theory^24^ and ray tracing analysis was developed for bulk and surface scattering in the case of microcrystalline silicon by Poruba.^25^ The model was originally developed for a layer on a smooth surface with roughness on only one side. For the purposes of this work, we recalculated the model for double-side roughness, and we treated more accurately some of its details. The recalculated version is labeled as *Poruba model (with star). Equations of different versions of the Poruba model are given in the Supporting Information. The main parameter of the model is the root-mean-square roughness (RMS). Finally, the simplest mathematical model of absorptance enhancement due to light trapping is the model of uniform extension of the path length by a constant δ. Then, absorptance following the Lambert–Beer law takes a simple form of eq 2

2 $$A≅{1-e}^{-\alpha \delta d}$$

Note that in this model we do not account for any internal reflections of light. The goal is to evaluate the potential of random roughness scattering and different scattering models from the point of insufficient absorptance in lead halide perovskite structures in the range from 500 to 800 nm.

## Theory

For illustration, the above-mentioned models are theoretically compared in Figure 1. As the model perovskite material, we chose the most basic methylammonium lead iodide CH_3_NH_3_PbI_3_ (MAPI). Optical constants of this material were determined from optical measurement of a layer prepared on a glass substrate. For the details of material preparation and optical properties determination refer to the Supporting Information. The simulated thickness was 500 nm. This represents the baseline case (single pass). In all cases, the loss from reflectance was neglected. For different models, we focused on the absorptance from 1.5 to 2.2 eV. Below the bandgap, we observed (*x*-axis is stretched here) an apparent absorption edge shift, and above the bandgap, we observed how the curve approached complete absorption. It can be seen that different models behave differently. Very efficient in approaching full absorption was extending (e.g., doubling) the photon path length (double pass, δ = 2), which was equivalent to implementing some strong geometrical light trapping/management (e.g., back reflector). On the other hand, the Yablonovitch limit does not reach full absorption above bandgap. The Yablonovitch limit was implemented here by gradually “switching on” from 5% to 100% by making a weighted average between the single pass and Yablonovitch limit. This represented the assumption that only part of the photons are scattered. The Yablonovitch limit had the strongest effect on the apparent absorption edge shift. The Poruba model was scaling with the value of RMS and was a bit similar to uniform photon path enhancement, but such enhancement would be limited to δ ≤ 2. Originally, the Poruba model was derived for single-side roughness, where the enhancement was a bit higher for higher roughness. As in our case, we mainly deal with layers deposited on the rough substrate, leading to roughness on both sides, we performed a new, more detailed derivation labeled by a star (*Poruba) for this case. However, the comparison showed that the original model can also be adapted to double-side roughness with good accuracy. For details about different versions and their comparison refer to the Supporting Information. To evaluate the effect on solar cell performance, the absorptance multiplied by solar radiation AM1.5G spectrum was integrated and relative photogeneration enhancement factors were evaluated; see graphical interpretation in Figure 2. If we try to approximately relate the path enhancement δ to RMS roughness in the *Poruba model, we obtain different trends for different layer thicknesses, see inset of Figure 2.

**Figure 1:**
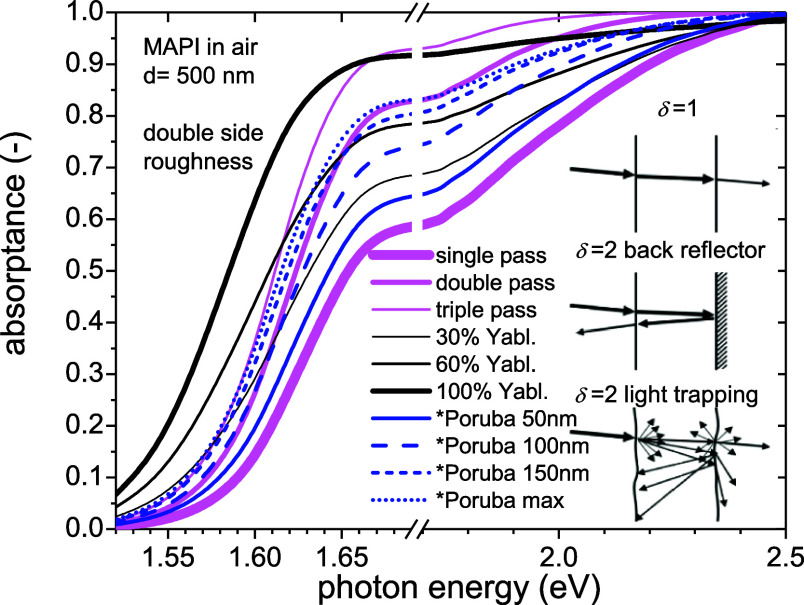
Absorptance curves (the reflectance is assumed to be zero) of CH_3_NH_3_PbI_3_ layers simulated by different analytical models for different intensities of the light trapping effect.

**Figure 2:**
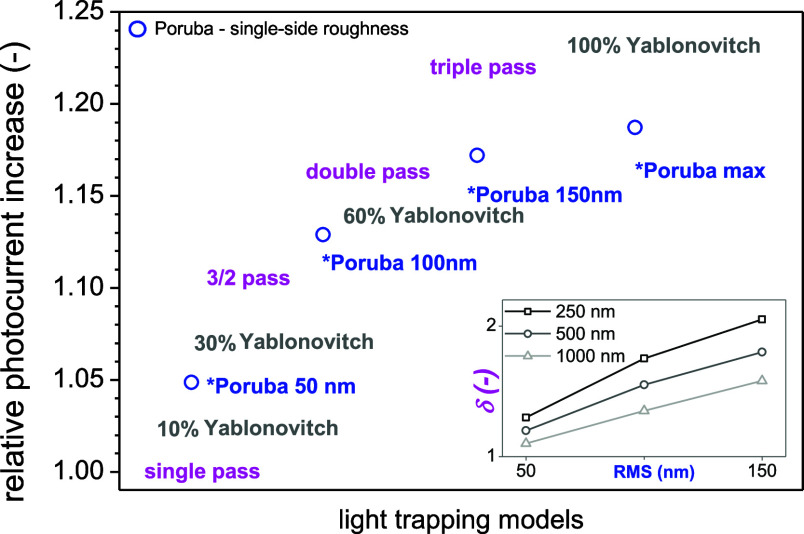
Graphical representation of relative photogeneration increases for different types of light trapping models for the CH_3_NH_3_PbI_3_ layer on glass with 500 nm thickness. The inset shows approximate relation between the Poruba model and uniform path enhancement.

We see that doubling the light path in a 500 nm thick layer of CH_3_NH_3_PbI_3_ results in more than 16% relative efficiency increase; tripling leads to 22% increase, which is almost as good as the maximum of the Yablonovitch limit with 23% increase, while the Poruba model can increase the photocurrent by only 16% relatively for double-side roughness. Interestingly, for single-side roughness the increase predicted by the Poruba model is up to 19%.

## Experimental Methods

### Transparent Conductive Oxide (TCO) Layers’ Preparation

The samples A, B, and C were prepared on corning glass using RF magnetron sputtering from 2 in. ZnO (99,99%) target with a substrate to target distance of 35 mm at RF power of 75, 150, and 175 W, respectively. Argon pressure was 2 × 10^–2^ Pa. Without any additional intentional heating, the substrate temperature was approximately 100 °C. Deposition time was 10 min.

### FA_0.9_Cs_0.1_PbI_3_ Material Preparation

The 1 M FA_0.9_Cs_0.1_PbI_3_ perovskite films were deposited from a precursor solution prepared by dissolving 0.9 mmol of FAI, 0.1 mmol of CsI, and 1 mmol of PbI_2_ in 1 mL of a mixed solvent consisting of DMF and DMSO in a 4:1 ratio. This precursor solution was continuously stirred at 60 °C for 1 h and then left to stir overnight. The resulting perovskite solution was then spin-coated onto the substrate, first at 1000 rpm for 10 s, followed by 5000 rpm for 30 s. During the second spin-coating step, 200 μL of ethyl acetate was dropped onto the spinning substrate 5 s before the end. Finally, the samples were annealed at 100 °C for 15 min. All fabrication steps were performed in a nitrogen-filled glovebox.

### CH_3_NH_3_PbI_3_ Material Preparation

Samples MAPI A and MAPI B were fabricated from precursor solutions containing 1 mmol of PbI_2_ and 1 mmol of CH_3_NH_3_I dissolved in 1 mL of DMF. Different amounts of MACl (2.5 wt % for MAPI A and 1.5 wt % for MAPI B) were added to these solutions. After stirring overnight, the solutions were spin-coated onto glass substrates at 4500 rpm for 40 s. The resulting films were then annealed at 100 °C for 3 min to create the CH_3_NH_3_PbI_3_·MACl layers. Once cooled to room temperature, the films were briefly exposed to CH_3_NH_2_ gas for about 2 s. After the gas was released from the films, the films were subjected to a final annealing at 150 °C for 10 min to produce high-quality CH_3_NH_3_PbI_3_ films. All the steps were performed in a nitrogen-filled glovebox. More details can be found in publication.^26^

Sample MAPI C was deposited on corning glass substrates from a precursor solution performed by dissolving 1.5 mmol of PbI_2_ and 1.5 mmol of MAI in 1.5 mL of solvent mixture of GBL and DMSO in a ratio of 3:2. This mixture was continuously stirred at 60 °C. The resulting perovskite solution was then spin-coated onto the substrate, first at 1000 rpm for 10 s and then at 5000 rpm for 30 s. During the second spin-coating step, 150 μL of chlorobenzene was dropped onto the spinning substrate 5 s before the end. Finally, the samples were annealed at 100 °C for 10 min. All the steps were performed in a nitrogen-filled glovebox.

## Characterizations

Photothermal deflection spectroscopy (PDS) measurements were performed by using a custom-built setup equipped with a 150 W Xe lamp and an Andor Kymera 328i spectrograph. The slit width was set to 1 mm, and 1:1 magnification focusing optics were employed. Fourier transform photocurrent spectroscopy (FTPS) measurements were conducted by using a Thermo Nicolet 8700 FTIR spectrometer equipped with an external tungsten light source, an external voltage source, and a Keithley 428 preamplifier. Scanning electron microscopy (SEM) was carried out using a TESCAN MAIA 3 operated at an accelerating voltage of 5 kV. Atomic force microscopy (AFM) was performed with a WiTec alpha300 SNOM system, utilizing the noncontact mode with Si probes. The angular distribution function (ADF) was obtained by using a custom-built optical setup. Full details of the measurement setups and calculation procedures are provided in the Supporting Information.

## Results

### Native Perovskite Surface Roughness

If we want to evaluate the absorptance enhancement, we have to know the absorption coefficient and refractive index of a single layer on glass (Figure S5 in Supporting Information). We therefore start with layers on glass. We prepared a set of 9 different layers of CH_3_NH_3_PbI_3_ on glass with varying thicknesses and varying crystallinity, in order to control surface roughness. The thickness categories were 160, 250, and 500 nm, and the grain size categories were S, M, and L as small/medium/large. For varying crystallinity, the previously developed recipe was used.^26^ Absorptance was then evaluated by measurement of Photothermal Deflection Spectroscopy (PDS). This measurement was performed in a liquid with a refractive index *n*_liquid_ = 1.25, which was close to 1, and does not considerably reduce the surface scattering compared to the case of air. In the models, however, this detail was considered. For accurate comparison between the models and the samples that exhibited a slight variation in the thicknesses, the interference fringes were first removed by dividing the measured absorptance by (1*R*)^27^ and the thickness variation was corrected by running the Lambert–Beer law back and forth to match the thickness of the respective category. The thicknesses were measured by cross-sectional SEM. For more details, refer to the Supporting Information. We tried to reproduce the behavior by simplest models, and we saw that already the model of uniform path length enhancement (δ > 1) reproduced all the experimental curves sufficiently well, see Figure 3. Remarkably, the values of uniform path length enhancements up to δ = 2.7 were observed. Referring to Figure 2, such a high value may be achieved only for samples with only one scattering surface in the Poruba model. Obviously, the question is how the true value of the absorption coefficient can be determined. We simply took the lowest value that we could observe, in the case of sample L500, the only one attributed to the path length enhancement of δ = 1.

**Figure 3:**
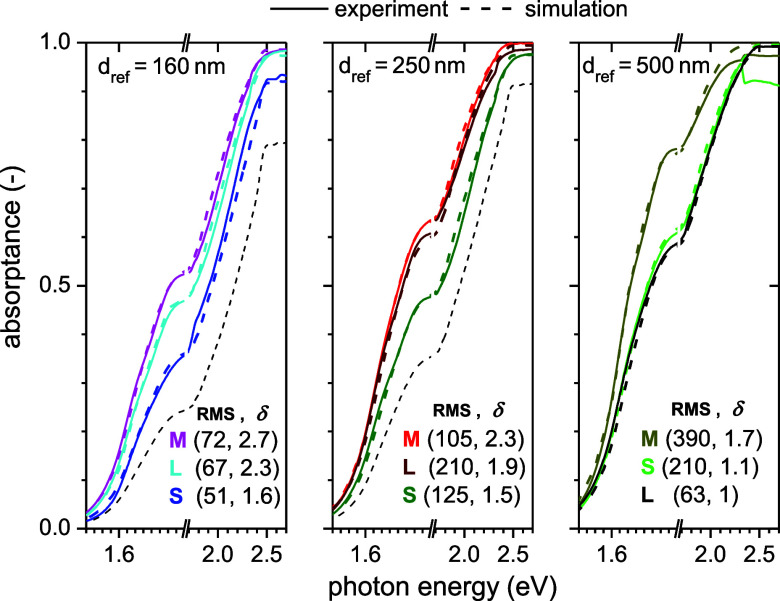
Absorptance of CH_3_NH_3_PbI_3_ layers on glass measured by PDS and recalculated to the same thicknesses (labeled *d*_ref_), fitted by the model (dashed line) with uniform path length enhancement (values of path length δ are given in brackets together with RMS roughness in nm).

For proving the absorptance enhancement independently from any simulations, we also measured the photocurrent spectrum between the two electrodes by Fourier-Transform Photocurrent Spectroscopy (FTPS), from the layer side and from the glass side. For more details, refer to the Supporting Information. It is well-known from the photocurrent methodology that the size of the actual illumination and detection area has to be considered. The scattered light in the spectral region around 1.5 eV, where the absorption coefficient was around 100 cm^–1^ had a penetration length of around 0.1 mm and could therefore travel parallel to the substrate. Like this, the illumination of the layer through the gap between the electrodes gave different results compared to the situation when a much larger area was illuminated from the glass side. The effect is schematically sketched in Figure S7. These effects of illumination and electrode geometry on the signal enhancement in low absorptance region were described extensively in relation to light trapping in thin films of microcrystalline silicon.^25,28^ In Figure 4, we compared FTPS spectra measured by illumination from the layer side (through the electrode gap) and by illumination from the glass side. In the graph, the ratio of the two curves is also shown, indicating enhancements of around 30%. The value was only approximate as the two spectra were normalized to each other at 1.7 eV. The purpose of this test was just to prove the existence of any absorption enhancement due to light trapping.

**Figure 4:**
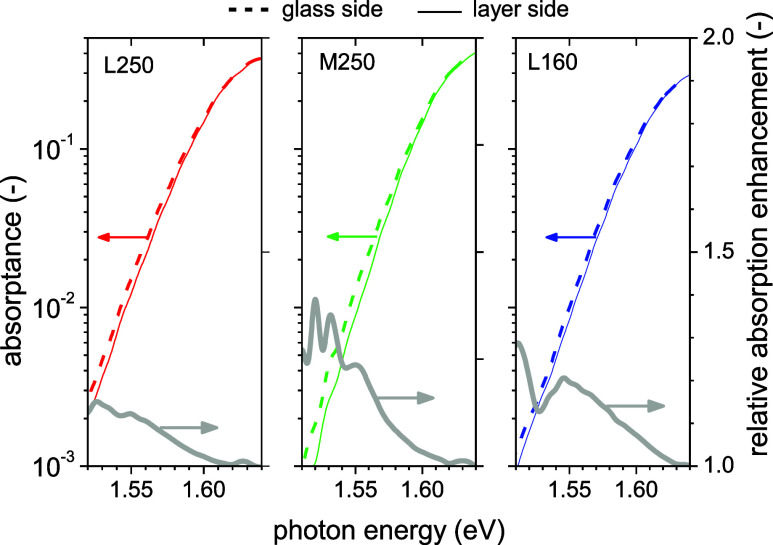
Simple proof of the light trapping by the observed difference between FTPS spectra measured by illumination from the glass side and a layer side (through the gap in the electrodes) for randomly chosen samples.

The effect of light trapping requires scattering either from some bulk features or from the surface. The light trapping enhancement δ can be compared to RMS values obtained from atomic force microscopy (AFM), see Figure S9 in the Supporting Information. RMS values and δ values are given in the legend of Figure 3. We see, however, that the correlation was not very good in this case. This means that the RMS as a single number was not a sufficient parameter for describing the light scattering effect. To find a better correlation, the angular distribution function (ADF) of the scattered light in the reflection mode from the layer side was measured with a red laser, see Figure 5. Although scattering in reality happens on the internal surface, while we can experimentally assess this only on the external surface, ADF can still serve for a relative comparison. In principle, the relation of internal and external ADF is possible using existing scattering models.^15^ The ADF functions were normalized to the laser intensity. Refer to the Supporting Information for further measurement details. The light scattering from the surface was increasing from sample L160 (lowest) to M500 (highest). Comparing with path length enhancement, we see that M160 had the highest δ value, which had the highest ADF curve among samples in 160 nm thickness category. On the other hand, the lowest δ was found for L500 that had the lowest ADF in the 500 nm thickness category. This means that not only ADF values but also the thickness has an effect on light trapping enhancement.

**Figure 5:**
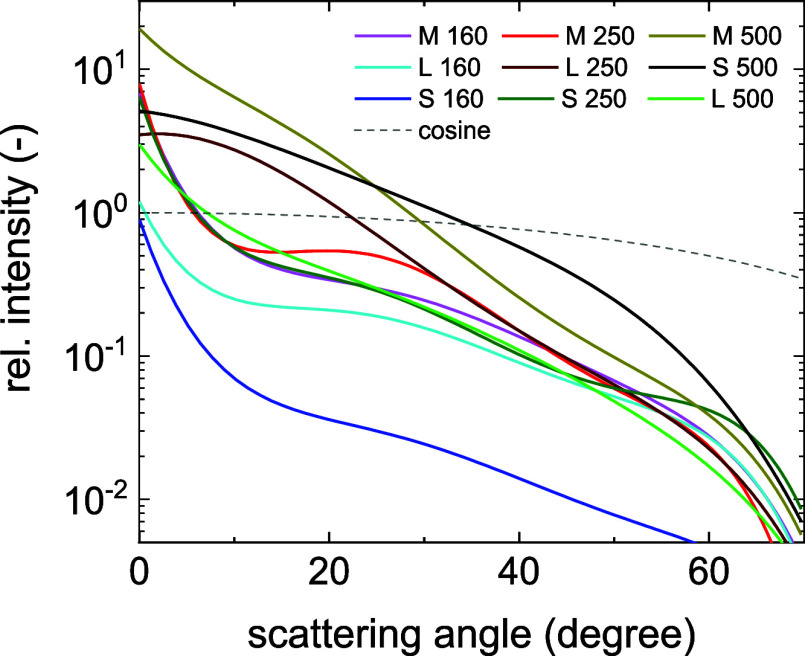
Angular Distribution Function of scattered light from CH_3_NH_3_PbI_3_ layers on glass measured in reflectance mode by a red laser.

### Nanorough and Microrough Substrates

While the native perovskite surface roughness in contact with air may exhibit remarkable absorptance enhancement, the effect of the interface with the substrate might be more important practically. Therefore, we tested the perovskite layers prepared on nanorough TCO substrates and on microrough glass substrates. Motivated by thin-film silicon technology,^29,30^ we first deposited various layers of ZnO by radiofrequency sputtering with varying power density leading to varying surface roughnesses (samples ZnO A, ZnO B, and ZnO C). The third sample (FTO) was a commercial SnO_2_:F U-type layer from Asahi Glass Company.^19,31^ Atomic force microscopy (AFM) images are provided in the Supporting Information. To increase the refractive index contrast, the layer of gold (100 nm) was coated on the top of the surfaces, mimicking the surface texturing of the back reflector.^16^ Perovskite layers were then deposited by spin coating on TCO and on glass for reference. Details about the layer preparation are provided in the Supporting Information. To avoid instability issues linked to methylammonium or mixed halide perovskites, the material of choice for experiments with different roughnesses was FA_0.9_Cs_0.1_PbI_3_. These layers were stable when deposited on TCO substrates. From the cross-sectional SEM images (inset of Figure S9), we can see that the roughness of the substrate had a negligible effect on the surface roughness of the perovskite layer. We measured the absorptance by PDS. The measurement was performed in a range where the absorptance in perovskite dominated over the absorptance in TCO or gold. The latter one can be revealed due to its almost constant absorptance contrasting with sharp absorption edge of perovskite. Spectra were corrected again by dividing by (1*R*). We tried to interpret the simulated curves in the framework of simple models and we found that a combination of the Yablonovitch model with a small contribution of uniform path enhancement (δ ≤ 1.3) was reproducing the results with good approximation, see Figure 6. Note that for a layer on a (thick) glass measured by PDS, the term ${\left(n_{amb}\right)}^{2}$ was set to *n*_glass_ *n*_liquid_ to account for the refractive index of the glass and the liquid. In the case of the gold layer, the factor 2 in the Yablonovitch limit was replaced by 4 and ${\left(n_{amb}\right)}^{2}$ was set to ${\left(n_{liquid}\right)}^{2}$ . Interestingly, unlike the native surface roughness, for these nanorough TCOs the Yablonovitch model is always necessary, and in the case of bare ZnO samples and FTO with gold coating, the uniform path enhancement does not apply at all. For the latter sample, even the complete Yablonovitch limit was achieved. The parameters are summarized in Table 1. The light trapping enhancement also correlated with the ADF functions shown in Figure 7.

**Figure 6:**
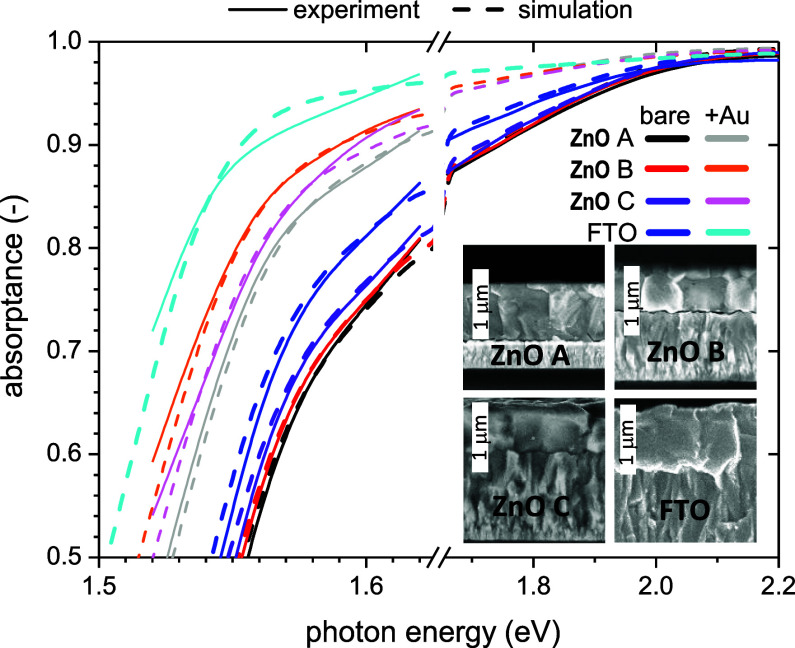
Absorptance of FA_0.9_Cs_0.1_PbI_3_ layers on top of TCO measured by PDS (without correction to varying thicknesses of the perovskite layer). Inset shows the cross-sectional SEM images (white scale bar represents 1 μm).

**Table 1:** Model Fit Parameters

	bare			with gold coating		
sample	RMS (nm)	enhancement δ	Yabl. contrib. (%)	RMS (nm)	enhancement δ	Yabl. contrib. (%)
ZnO A	26 ± 6	1	10	26 ± 6	1.3	50
ZnO B	34 ± 10	1	15	32 ± 9	1.2	75
ZnO C	50 ± 17	1	25	61 ± 17	1.2	65
FTO	173 ± 42	1.15	35	150 ± 50		100
glass 0	8 ± 2	1		10 ± 2	1.4	50
glass A	112 ± 21	1.05		266 ± 53	1.3	70
glass B	80 ± 21	1.2		57 ± 13	1.4	60
glass F	220 ± 110	1.6	45	500 ± 100	1.4	75

**Figure 7:**
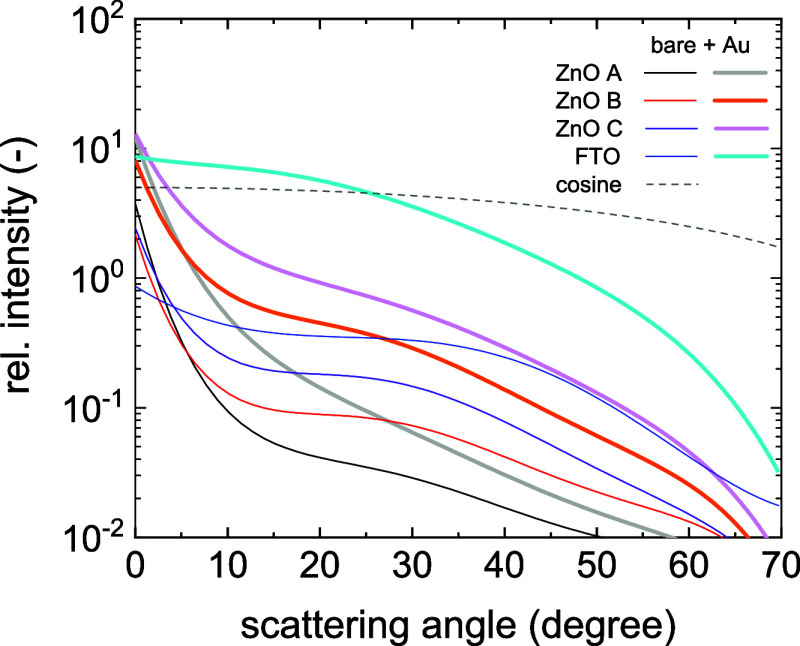
Angular Distribution Function of scattered light from nanorough TCO layers with and without gold coating, measured by a red laser.

In parallel with the nanoroughness obtained by textured TCO, we also investigated a microrough surfaces of glass prepared by very simple mechanical methods. Corning microscopic slides CLS294775 × 50 were used in different treatments, named glass 0, A, B, and F. Glass 0 was without any treatment, glass A and glass B were grinded by sandpaper with density 2000 and 1200, respectively, and glass F was the blasted (frosted) area of the microscope slide. Resulting PDS spectra are shown in Figure 8. From the SEM images (inset of Figure 8), we see that the perovskite layer cannot conformally coat the glass F substrate, and the thickness therefore does not have a physical meaning. For the calculations, however, we assumed a value of 500 nm. We again reproduced the results by the combination of uniform path enhancement (δ > 1) with the Yablonovitch model. For glasses without a gold coating, except glass F, a small (δ ≤ 1.2) path enhancement alone described the light trapping effect. For glass F and the use of gold coating, the Yablonovitch model must be included but never fully as in the case of nanorough surfaces. The parameters are summarized in Table 1. The light trapping enhancement also correlated with the ADF functions as shown in Figure 9.

**Figure 8:**
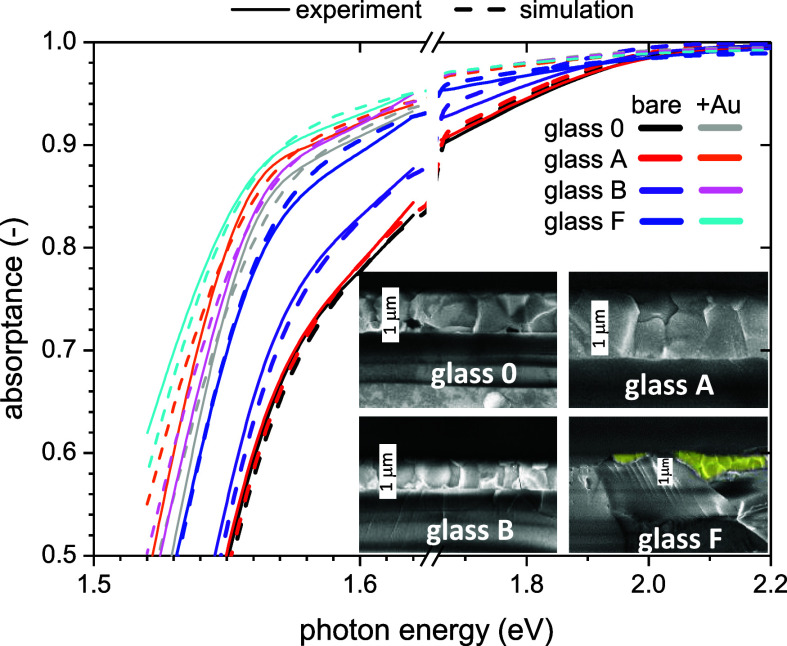
Absorptance of FA_0.9_Cs_0.1_PbI_3_ layers on top of glass substrates with different roughnesses, measured by PDS and corrected to the thickness variations. The inset shows the cross-sectional SEM images (white scale bar represents 1 μm, in the case of glass F the perovskite layer is highlighted by yellow color).

**Figure 9:**
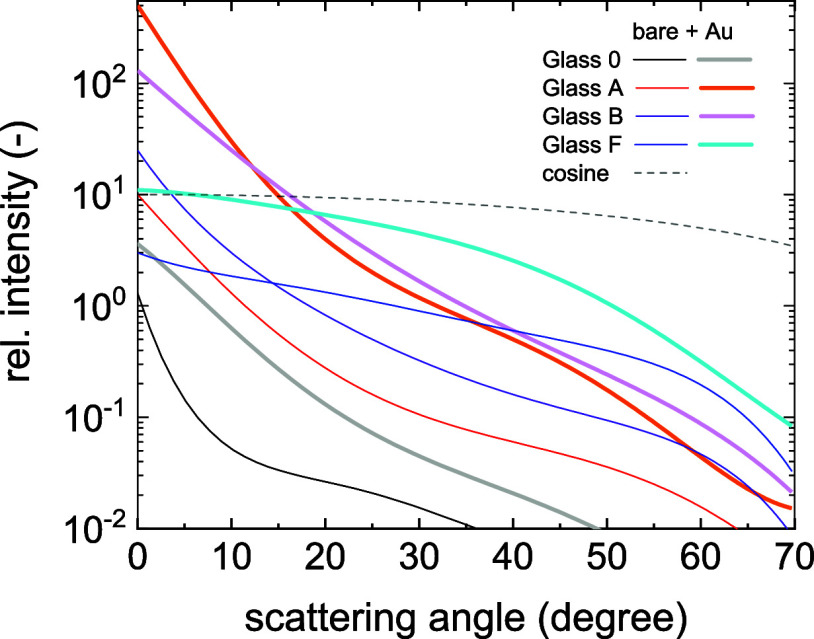
Angular Distribution Function of scattered light from microrough glass substrates with and without gold coating, measured by a red laser.

## Conclusions

In conclusion, this work studies the role of random texture scattering in enhancing absorptance particularly between 500 and 800 nm in hybrid halide perovskite layers. Our findings reveal that nanorough textured substrates offer limited gains due to low refractive index contrast. This can be emphasized by introducing a metal (Au) coating that considerably improves the light trapping, tending to follow the Yablonovitch limit, enhancing mainly medium absorbing photons. Microrough substrates, produced by mechanical treatment of glass, led to enhancement that is more tending to uniform optical thickness enhancement. In contrast with that is the native surface roughness of perovskite layers in air, which behavior follows uniform optical thickness completely, effectively extending the optical path length several times. Experimentally, this can be revealed by comparing photocurrent spectroscopy between coplanar contacts, measured from the film and substrate side. The uniform optical thickness enhancement is a good first approximation to a much-sophisticated Poruba model that is based on scalar scattering theory, and in agreement with this model, the optical path enhancement is stronger in thinner layers and layers with only one scattering surface. Scalar scattering theory, taking RMS roughness as a parameter, represents a good base for modeling light trapping in solar cells as well as for evaluation of optical constants; however, the RMS parameter does not correlate to the one obtained from AFM measurements. This work highlights the importance of light trapping enhancement from the perspective of potential in improving perovskite photovoltaic devices.

## Supplementary Material



## Notes

Details of Poruba’s model calculations for surface roughness, considering one side and both sides’ scattering cases; setups for PDS, FTPS, and ADF measurements; SEM images to characterize the thickness and surface morphology of CH_3_NH_3_PbI_3_ perovskite layers; and AFM images to evaluate the surface roughness of CH_3_NH_3_PbI_3_ perovskite layers, nanorough TCO substrates, and microrough glass substrates (PDF)
