# Supplementary material for: The Role of Random Texture Scattering on the Absorptance Enhancement in Halide Perovskite Layers
Source: ACS Appl Mater Interfaces. 2025 Aug 20;17(35):49986–92. doi: 10.1021/acsami.5c09757 (PMC12412110; doi:10.1021/acsami.5c09757)
Supplement: Supplementary file 1 [file am5c09757_si_001.pdf]

SUPPORTING INFORMATION

Role of Random Texture Scattering on the Absorptance Enhancement in Halide Perovskite Layers

*Meng-Hsueh Kuo[1,2], Branislav Dzurňák[1], Neda Neykova[1,2], Lucie Landová[1,2], Ivana Beshajová Pelikánová[1], Zdeněk Remeš[2], Chih-Yu Chang[3], Stefaan De Wolf[4] and *Jakub Holovský[1,2]

[1] Centre for Advanced Photovoltaics, Faculty of Electrical Engineering, Czech Technical University in Prague, Technická 2, 16627 Prague, Czech Republic
[2] Institute of Physics, Czech Academy of Sciences, Cukrovarnická 10, 16200 Prague, Czech Republic
[3] Department of Materials Science and Engineering, National Taiwan University of Science and Technology, Taipei, 10607 Taiwan (R.O.C.)
[4] King Abdullah University of Science and Technology (KAUST), KAUST Solar Center (KSC), Thuwal, 23955-6900, Saudi Arabia

*Corresponding authors: kuomengh@fel.cvut.cz, jakub.holovsky@fel.cvut.cz

Calculations

1) Poruba's model for roughness on one side [1]:
Complex refractive index N can be defined from refractive index n and absorption coefficient α as follows (i is imaginary unit and λ is wavelength):

$$N = n + \mathrm{i}\frac{\alpha\lambda}{4\pi} \qquad (1)$$

Fresnel intensity coefficients for perpendicular incidence for reflectance R and transmittance T (n_1 is refractive index of medium on the first, incoming side, n_2 is refractive index of medium on the second, transmitting side) are defined as follows:

$$t_{12} = \frac{2n_1}{n_1+n_2}\ , T_{12} = \frac{n_2}{n_1}|t_{12}|^2 \qquad (2)$$

$$r_{12} = \frac{n_1-n_2}{n_1+n_2}\ , R_{12} = |r_{12}|^2 \qquad (3)$$

The amount of specularly transmitted or reflected light is obtained by multiplying Fresnel intensity coefficients by scalar scattering theory [2] scattering factors (σ is RMS roughness, n_f is refractive index of film, n_a is the refractive index of ambient, φ is the angle of incidence that for trapped light can be approximated by π/n_f):

$$S_T = \exp\left[-\left(\frac{2\pi(n_f-n_a)\sigma}{\lambda}\right)^2\right] \qquad (4)$$

$$S_{R,0} = \exp\left[-\left(\frac{4\pi n_f\sigma}{\lambda}\right)^2\right] \qquad (5)$$

$$S_R = \exp\left[-\left(\frac{4\pi n_f\sigma\cos\varphi}{\lambda}\right)^2\right] \cong \exp\left[-\left(\frac{4\pi n_f\sigma\cos(\pi/n_f)}{\lambda}\right)^2\right] \qquad (6)$$

The light absorbed directly without scattering event is (d is the film thickness):

$$A_{dir} = S_T(1-e^{-\alpha d}) \qquad (7)$$

Leakage of photons from beam (non-scattered) into scattered light through scattering at the surface and at the hitting the surface after reflection (of non-scattered beam) from the back side of the film is (indices $f$and s mean film and substrate, respectively):

$$P_0 = (1-S_T) + S_T R_{fs} e^{-2\alpha d} R_{fa}(1-S_{R0}) \qquad (8)$$

Probability of light scattering into escape cone, for Lambertian case is:

$$P_{esc} = (n_s/n_f)^2 \qquad (9)$$

The multiplication factor of average optical path increase for Lambertian distribution follows. Angles $\beta$and γ are the integration limits that are linked to escape cone:

$$Y_\beta^\gamma = 2\frac{\int_\beta^\gamma \sin\varphi\, d\varphi}{\int_\beta^\gamma \sin\varphi\cos\varphi\, d\varphi} \qquad (10)$$

The factor "2" is means according to [1] "twofold path because of the total internal reflection at the film–substrate smooth interface".

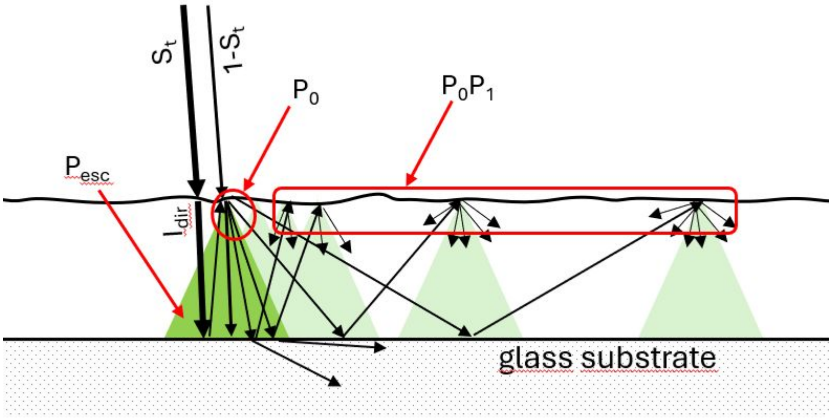

Figure S1: Illustration of the different contributions into Poruba model.

The portion of absorbed light from the scattered light (inside and outside of the escape cone) between first and second scattering event (γ is the angle of escape cone into substrate for which the condition is $\sin(\gamma) = n_s/n_f$):

$$A_1 = (1 - P_{esc})[1 - \exp\left(-Y_{\gamma}^{\pi/2}\alpha d\right)] + P_{esc}[1 - \exp\left(-Y_0^{\gamma}\alpha d\right)] \qquad (11)$$

The relative intensity reduction between the first and second scattering event is:

$$P_1 = (1 - P_{esc})\exp\left(-Y_{\gamma}^{\pi/2}\alpha d\right)] \qquad (12)$$

The portion of light (scattered and non-scattered, inside and outside of escape cone) absorbed between first and second scattering event (γ is the angle of escape cone into substrate for which the condition is $\sin(\gamma) = n_s/n_f$):

$$A_2 = [(1 - P_{esc})(1 - S_r) + S_r)][1 - \exp\left(-Y_{\gamma}^{\pi/2}\alpha d\right)] + P_{esc}(1 - S_r)[1 - \exp\left(-Y_0^{\gamma}\alpha d\right)][1 + R_{fs}\exp\left(-Y_0^{\gamma}\alpha d\right)] \qquad (13)$$

The relative intensity reduction between each two consecutive scattering events of light previously scattered outside escape cone:

$$P_2 = [(1 - P_{esc})(1 - S_r) + S_r)]\exp\left(-Y_{\gamma}^{\pi/2}\alpha d\right) \qquad (14)$$

Altogether, the intensity of absorbed light is the sum of an infinite row (in the term expressing sum of infinite row, all reflectances are unity):

$$A = A_{dir} + P_0 A_1 + P_0 P_1 A_2 \frac{1}{1 - P_2} \qquad (15)$$

2) Poruba's model adapted to roughness on both sides

The simplest way of accounting for the same type of roughness on both sides of the layer is by removing "2" from the multiplication factor of average optical path increase for Lambertian distribution – the change indicated by star (*):

$$Y_{\beta}^{*\gamma} = \frac{\int_{\beta}^{\gamma} sin\,\varphi\, d\varphi}{\int_{\beta}^{\gamma} sin\,\varphi \cos\varphi\, d\varphi} \qquad (16)$$

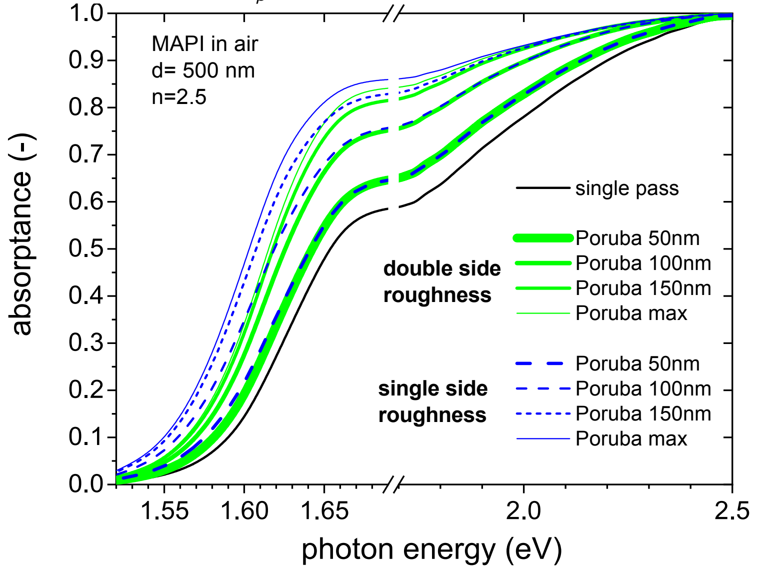

Figure S2: Illustration of the difference between the original Poruba model for single side roughness and its simple modification for double side roughness. We see that for low to medium roughness the second roughness has no big effect, but for larger roughness the effect is negative.

3) Poruba's model adapted to roughness on both sides and multiple reflectances everywhere:
More accurate way is to build the model again from scratch with accounting scattering on both sides and multiple reflections everywhere summed as infinite rows. Definitions of N, t_{12}, r_{12} , S_T, $S_{R,0}$ remain the same Y_{β}^{γ} will be replaced by $Y_{\beta}^{*\gamma}$. We do assume the surface roughness on both interfaces, but we will assume the same parameter σ for both interfaces. Interestingly, according to scalar scattering theory [2] the coefficient $S_{R,0}$ depends only on the refractive index of the film, therefore remains the same for both interfaces. However, for better accuracy the angular dependence of S_R should be accounted by replaced it by $S_{R,1}$ for photons inside escape cone and $S_{R,2}$ for photon outside escape cone. We define the angle of escape cone based on the media with higher refractive index, which is the one of the substrate (n_s). We stick to γ for which the condition is $\sin(\gamma) = n_s/n_f$: The average angles of light inside and outside of the escape cone for Lambertian distribution, calculated from $\int_{\beta}^{\gamma} \varphi sin\varphi \cos\varphi d\varphi / \int_{\beta}^{\gamma} sin\varphi \cos\varphi d\varphi$, are 24° and 51° respectively.

$$S_{R,1} = \exp\left[-\left(\frac{4\pi n_f \sigma \cos 24°}{\lambda}\right)^2\right] \qquad (17)$$

$$S_{R,2} = \exp\left[-\left(\frac{4\pi n_f \sigma \cos 51°}{\lambda}\right)^2\right] \qquad (18)$$

The light absorbed directly without scattering event is (d is the film thickness) is now corrected to the multiple reflectances. The contributions from each reflectance are calculated as Poynting's vectors rather than electromagnetic field vectors. The reason is that unfortunately, the scalar scattering theory is developed for Poynting's vector and combining it with field calculations is not possible as there is already a loss of information about phase.

$$A^*_{dir} = S_T(1-e^{-\alpha d}) + S_T\, e^{-\alpha d} R_{fs} S_{R,0}(1-e^{-\alpha d}) + S_T\, e^{-2\alpha d} R_{fs}\, S^2_{R,0} R_{fa}(1-e^{-\alpha d})$$
$$+\ S_T\, e^{-3\alpha d} R^2_{fs}\, S^3_{R,0} R_{fa}(1-e^{-\alpha d})\ +\ S_T\, e^{-4\alpha d} R^2_{fs} S^4_{R,0} R^2_{fs}(1-e^{-\alpha d}) + \dots$$

$$= S_T(1-e^{-\alpha d})\{(1+\ e^{-\alpha d} R_{fs} S_{R,0})\,(1+\ e^{-2\alpha d} R_{fs}\, S^2_{R,0} R_{fa} +\ e^{-4\alpha d} R^2_{fs} S^4_{R,0} R^2_{fs} + \dots)\}$$

$$= S_T(1-e^{-\alpha d})\frac{1+e^{-\alpha d} R_{fs} S_{R,0}}{1-\ e^{-2\alpha d} R_{fs}\, S^2_{R,0} R_{fa}} \qquad (19)$$

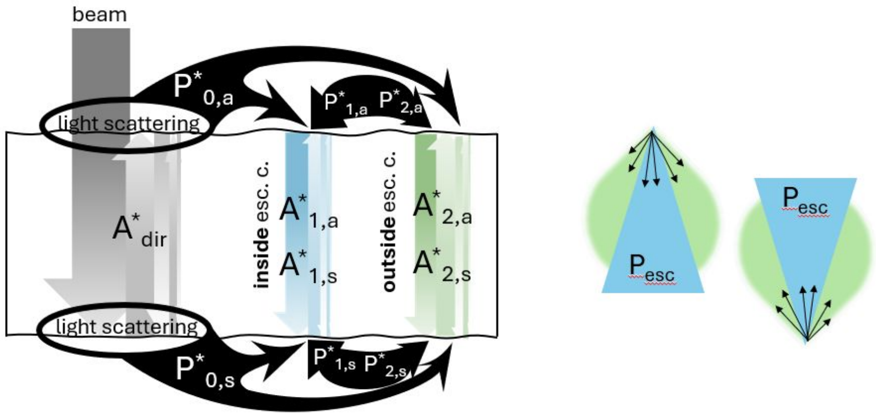

Figure S3: Illustration of the different contributions into recalculated Poruba model

Leakage of photons from beam (non-scattered) into scattered light through scattering at the front surface and after infinite reflections inside the layer (indices $f$and s mean film and substrate, respectively):

$$P^*_{0,a} = (1-S_T) +\ S_T R_{fs} S_{R,0} R_{fa} e^{-2\alpha d}(1-S_{R0}) + S_T R^2_{fs} S^3_{R,0} R^2_{fa} e^{-4\alpha d}(1-S_{R,0})$$
$$\dots\ = (1-S_T) + \frac{S_T R_{fs} S_{R,0} R_{fa} e^{-2\alpha d}(1-S_{R,0})}{1-\ e^{-2\alpha d} R_{fs}\, S^2_{R,0} R_{fa}} \qquad (20)$$

Leakage of photons from beam (non-scattered) into scattered light through scattering at the back surface and after infinite reflections inside the layer:

$$P^*_{0,s} =\ S_T R_{fs} e^{-\alpha d}(1-S_{R,0}) + S_T R^2_{fs} S^2_{R,0} R_{fa} e^{-3\alpha d}(1-S_{R,0}) + \dots$$
$$= \frac{S_T R_{fs}(1-S_{R,0})\ e^{-\alpha d}}{1-\ e^{-2\alpha d} R_{fs}\, S^2_{R,0} R_{fa}} \qquad (21)$$

Part of the light is scattered into escape cone, that is given by the refractive index of surrounding media. We take the media with higher refractive index, which is the one of the substrate (n_s). For Lambertian case the probability of scattering into escape cone is:

$$P_{esc} = (n_s/n_f)^2 \qquad (22)$$

Then probability that the light after reflection on rough surface remains in the escape cone:

$$Q_{R,1} = S_{R,1} + (1-S_{R,1})P_{esc} \qquad (23)$$

The probability that the light after reflection on rough surface remains outside escape cone:

$$Q_{R,2} = S_{R,2} + (1 - S_{R,2})(1 - P_{esc}) \qquad (24)$$

Analogically with $P^*_{0,s}$, leakage of photons from light scattered from inside the escape cone (blue in Figure S3) to the light outside the escape cone (green in Figure S3), through scattering at front surface $P^*_{2,a}$, taking into account infinite number of reflections inside the layer and contributions from both $P^*_{0,a}$ and $P^*_{0,s}$, can be written as follows:

$$P^*_{2,a} = (1 - P_{esc})\big(1 - S_{R,1}\big)P_{esc}\frac{P^*_{0,s}R_{fa}e^{-Y_0^{*\gamma}\alpha d} + P^*_{0,a}R_{fs}Q_{R,1}R_{fa}e^{-2Y_0^{*\gamma}\alpha d}}{1 - e^{-2Y_0^{*\gamma}}R_{fs}\,Q^2_{R,1}R_{fa}} \qquad (25)$$

Analogically, the same leakage but through scattering at back surface $P^*_{2,s}$, can be written as:

$$P^*_{2,s} = (1 - P_{esc})\big(1 - S_{R,1}\big)P_{esc}\frac{P^*_{0,a}R_{fs}e^{-Y_0^{*\gamma}\alpha d} + P^*_{0,s}R_{fs}Q_{R,1}R_{fa}e^{-2Y_0^{*\gamma}\alpha d}}{1 - e^{-2Y_0^{*\gamma}}R_{fs}\,Q^2_{R,1}R_{fa}} \qquad (26)$$

Analogically, leakage of photons from light scattered from outside the escape cone (green in Figure S3) to the light inside the escape cone (blue in Figure S3), through scattering at front surface $P^*_{1,a}$, can be written as follows (Note that now the reflectances term vanish as we deal with total reflectances):

$$P^*_{1,a} = (1 - P_{esc})\big(1 - S_{R,2}\big)P_{esc}\frac{P^*_{0,s}e^{-Y_\gamma^{*\pi/2}\alpha d} + P^*_{0,a}Q_{R,2}e^{-2Y_\gamma^{*\pi/2}\alpha d}}{1 - e^{-2Y_\gamma^{*\pi/2}}\,Q^2_{R,2}} \qquad (27)$$

Analogically, the same leakage but through scattering at back surface $P^*_{1,s}$, can be written as:

$$P^*_{1,s} = (1 - P_{esc})\big(1 - S_{R,2}\big)P_{esc}\frac{P^*_{0,a}e^{-Y_\gamma^{*\pi/2}\alpha d} + P^*_{0,s}Q_{R,2}e^{-2Y_\gamma^{*\pi/2}\alpha d}}{1 - e^{-2Y_\gamma^{*\pi/2}}\,Q^2_{R,2}} \qquad (28)$$

Now, knowing all the leakages from beam light and between the light inside and outside escape cone, we can write already the equation of total absorptance:

$$A^* = A^*_{dir} + (P^*_{0,a}P_{esc} + P^*_{1,a})A^*_{1,a} + (P^*_{0,s}P_{esc} + P^*_{1,s})A^*_{1,s} + \big[P^*_{0,a}(1 - P_{esc}) + P^*_{2,a}\big]A^*_{2,a} + \big[P^*_{0,s}(1 - P_{esc}) + P^*_{2,s}\big]A^*_{2,s} \qquad (29)$$

The last step is to calculate the terms $A^*_{1,a}$, $A^*_{1,s}$, $A^*_{2,a}$, $A^*_{2,s}$. The term $A^*_{1,a}$ determines the absorptance of light inside escape cone that is coupled-in at front interface:

$$A^*_{1,a} = (1 - e^{-Y_0^{*\gamma}\alpha d}) + e^{-Y_0^{*\gamma}\alpha d}R_{fs}Q_{R,1}\,(1 - e^{-Y_0^{*\gamma}\alpha d}) + e^{-2Y_0^{*\gamma}\alpha d}R_{fs}\,Q^2_{R,1}R_{fa}(1 - e^{-\alpha Y_0^{*\gamma}d}) + e^{-3Y_0^{*\gamma}\alpha d}R^2_{fs}\,Q^3_{R,1}R_{fa}(1 - e^{-Y_0^{*\gamma}\alpha d}) + \ldots$$

$$= (1 - e^{-Y_0^{*\gamma}\alpha d})\left\{1 + e^{-Y_0^{*\gamma}\alpha d}R_{fs}Q_{R,1} + e^{-2Y_0^{*\gamma}\alpha d}R_{fs}Q^2_{R,1}R_{fa} + e^{-3Y_0^{*\gamma}\alpha d}R^2_{fs}Q^3_{R,1}R_{fa} + \ldots\right\}$$

$$= (1 - e^{-Y_0^{*\gamma}\alpha d})\frac{1+e^{-Y_0^{*\gamma}\alpha d}R_{fs}Q_{R,1}}{1-e^{-2Y_0^{*\gamma}\alpha d}R_{fs}Q_{R,1}^2R_{fa}} \quad (30)$$

Analogically, the term $A_{1,s}^*$ determines the absorptance of light inside escape cone that is coupled-in at back interface:

$$A_{1,s}^* = (1 - e^{-Y_0^{*\gamma}\alpha d})\frac{1+e^{-Y_0^{*\gamma}\alpha d}R_{fa}Q_{R,1}}{1-e^{-2Y_0^{*\gamma}\alpha d}R_{fs}Q_{R,1}^2R_{fa}} \quad (31)$$

The term $A_{2,a}^*$ determines the absorptance of light outside escape cone that is coupled-in at front interface:

$$A_{2,a}^* = (1 - e^{-Y_\gamma^{*\pi/2}\alpha d})\frac{1+e^{-Y_\gamma^{*\pi/2}\alpha d}Q_{R,2}}{1-e^{-2Y_\gamma^{*\pi/2}\alpha d}Q_{R,2}^2} \quad (32)$$

The term $A_{2,s}^*$ determines the absorptance of light outside escape cone that is coupled-in at back interface, but is the same as $A_{2,a}^*$, so:

$$A_{2,s}^* = A_{2,a}^* \quad (33)$$

Comparison between the two latter approaches in Figure S4. We see that there is a constant trend of simpler model to slightly overestimate the absorptance, but the model is still valid.

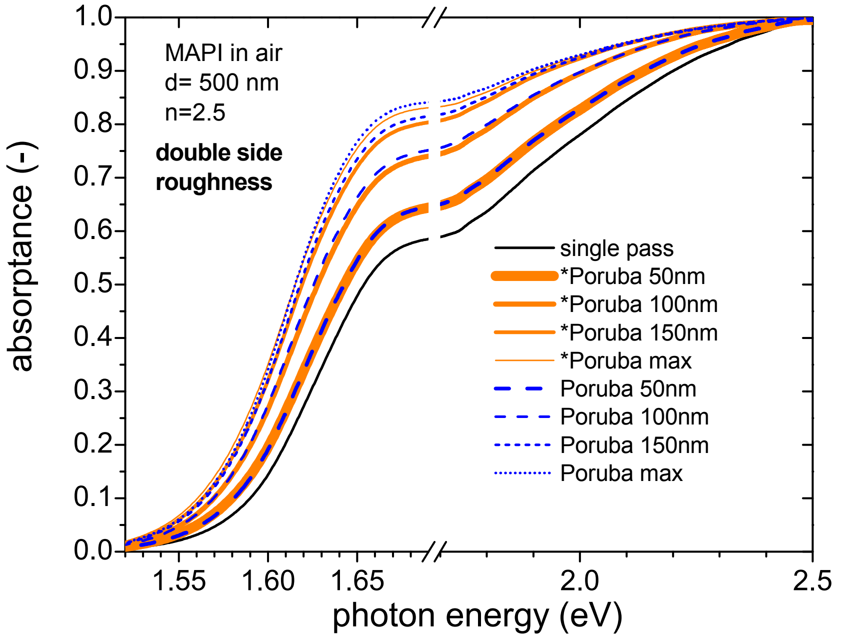

Figure S4: Illustration of the difference between the original Poruba model adapted to double side roughness and more accurate version (*Poruba).

Photothermal Deflection Spectroscopy measurements

The Photothermal Deflection Spectroscopy measurements were performed by a home-made setup equipped with 150W Xe lamp and Andor Kymera 328i. Slits were set to 1 mm. Focusing optics with magnification 1:1 was used. Combination of grating number of grooves and slit widths gave theoretical resolution

$$\frac{\Delta E}{E} \approx 0.01 \qquad (34)$$

, where E was photon energy. Real resolution, according to spectral linewidth measurements, was

$$\Delta E/E \leq 0.02. \qquad (35)$$

As a thermal sensitive liquid, Flutec PP1 was used. Refractive index was 1.25. Simultaneously, transmittance and reflectance were measured by integration spheres in front and behind the cuvette (not directly in front or behind the sample), cuvette internal dimensions were 10 x10 mm. Absorptance from PDS effect was absolutely scaled according to $1 - R - T$ measured by integrating spheres. Absorption coefficient was then evaluated from absorptance/transmittance ratio from a smooth sample on glass according to equations from ref. [3]. For other purposes of absorptance comparison the simple equation is assumed to be

$$A \cong 1 - \exp\left(-\alpha \delta d\right). \qquad (36)$$

More accurately, the equation reads

$$A_{exp} \cong (1 - R_0)\left[1 - \exp\left(-\alpha \delta d_{exp}\right)\right] \qquad (37)$$

, where R_0 is the reflectance on the first surface, d_{exp} is actual thin-film thickness. In high absorption region, the R_0 reflectance equal to the experimentally observed reflectance of the real stack, but for the purposes of this study we assume $R_0 \cong R$ everywhere. From experimentally obtained A_{exp} we calculated absorptance A that is corrected to reflectance effects and corrected to thickness variations as

$$A \cong 1 - \exp\left[d/d_{exp} * \ln(1 - A_0/(1 - R))\right]. \qquad (38)$$

Because the PDS measurement is not taken from one point, but rather from a line that is approximately 8 mm long, the thickness variations are often averaged. This contrasts with determining the thickness from scanning electron microscopy of a cross-section image, where random points are taken and the results might be misleading. Therefore, the thickness correction has to be done only in necessary cases.

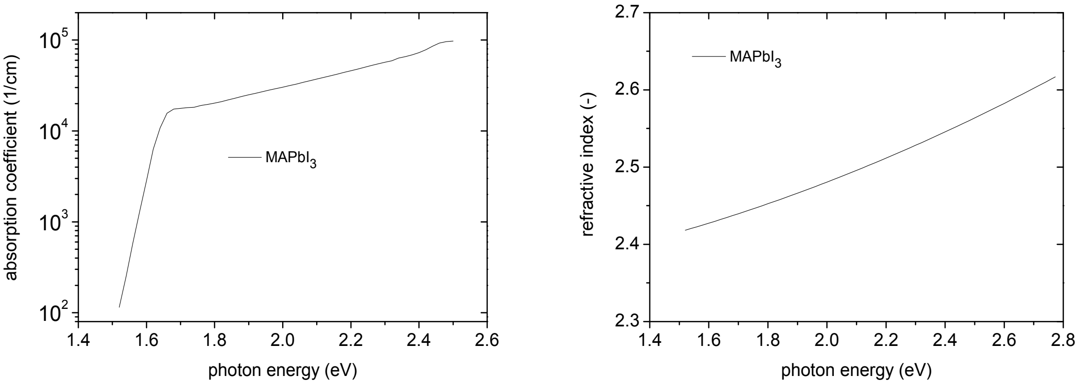

Figure S5: Absorption coefficient and refractive index of MAPI perovskite layer obtained from PDS measurement and numeric fitting procedure.

Fourier Transform Photocurrent Spectroscopy measurements

On the samples dedicated for FTPS measurements, the planar electrodes were prepared by evaporation gold trough mechanical mask. The contact distance is 0.5 mm. The electrode pattern is in Figure S6.

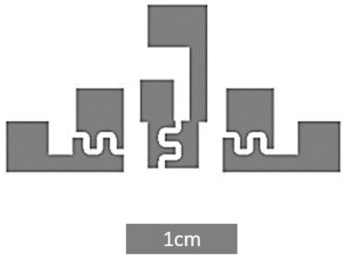

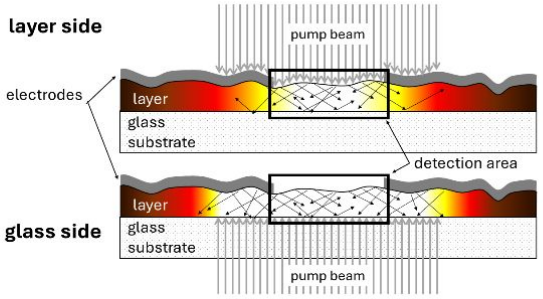

Figure S6: electrode pattern for FTPS measurements.

Figure S7: Sketch of the FTPS sample arrangement and the effect of the illumination direction and the role of trapped light. In the case of glass side the long travelling photons contribute more to the measurement.

FTPS was performed by FTIR Thermo Nicolet 8700 equipped with external tungsten light source and external voltage source and pre-amplifier Keithley 428. Voltage bias 10 V was applied, giving DC current in the range of ~10 nA. Preamplification 10^8 V/A was applied. Infrared glass optical filter RG 780 from Thorlabs was used to suppress visible part of the spectrum and to collect sub-bandgap part of the spectrum. Scan speed velocity was 0.16 cm/s leading to modulation frequency around 4 kHz. Frequency dependence was corrected based on the measurements at twice and three times higher modulation frequencies.

Scanning Electron Microscopy measurements

The thicknesses of the perovskite thin films were determined from sample cross sections using scanning electron microscope MAIA 3, TESCAN at voltage of 5 kV.

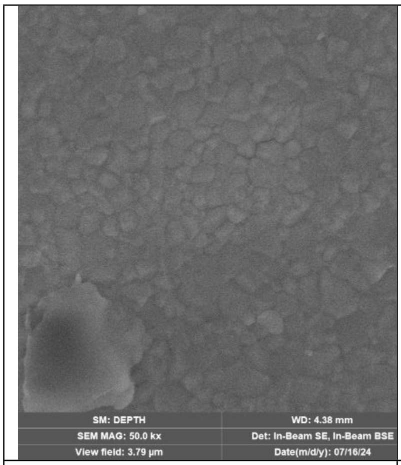

S 160 (int. no. 2024 23a)

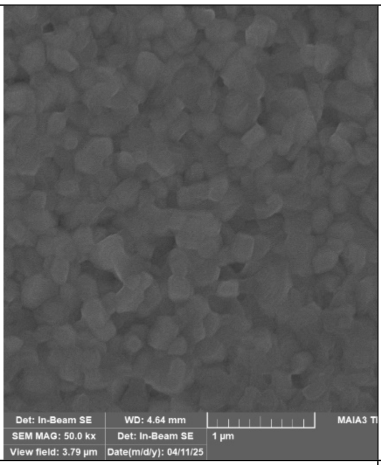

M 160 (int. no. 2025 I3)

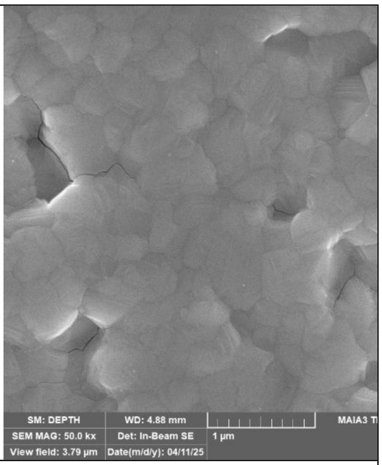

L 160 (int. no. 2025 C3)

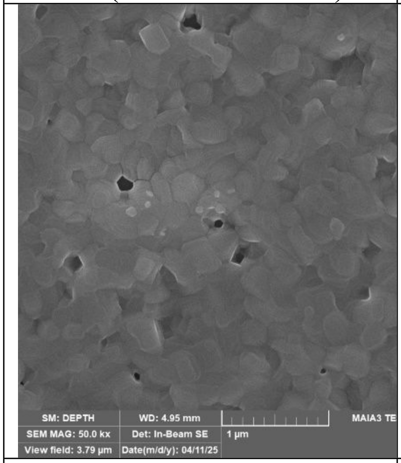

S 250 (int. no. 2025 G3)

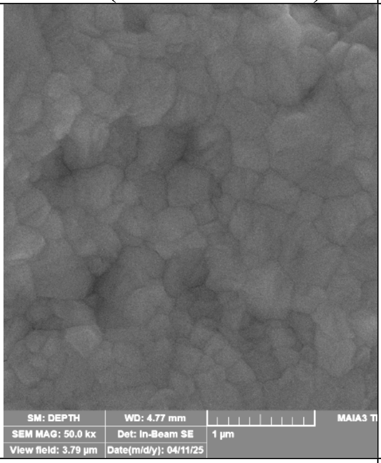

M 250 (int. no. 2025 B3)

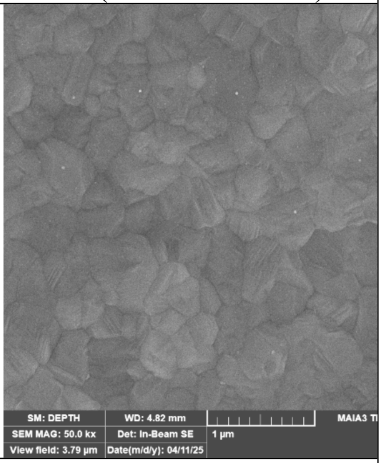

L 250 (int. no. 2025 A3)

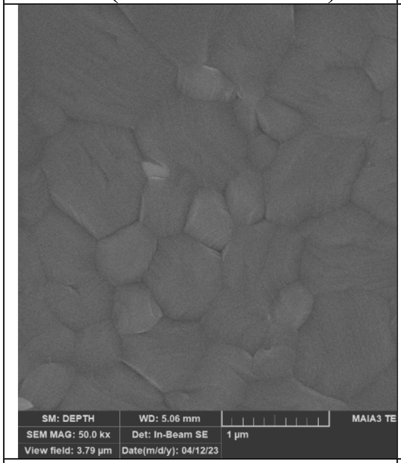

S 500 (int. no. 2024 C2)

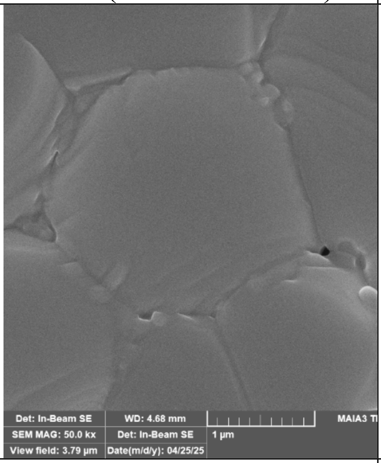

M 500 (int. no. 2025 D15)

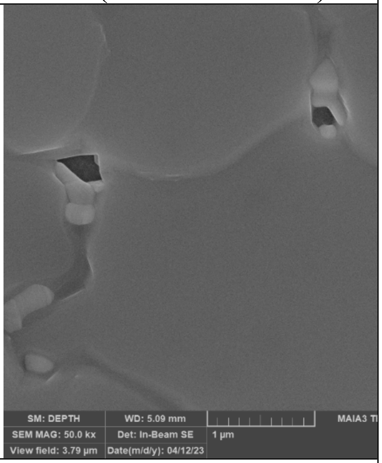

L 500 (int. no. 2024 E1)

Figure S8: SEM images of $CH_3NH_3PbI_3$ samples.

Atomic Force Microscopy

Surface roughness was measured by AFM using WiTec alpha300 SNOM system utilizing non-contact AFM method with Si probes. Measured sample area was 5 × 5 µm.

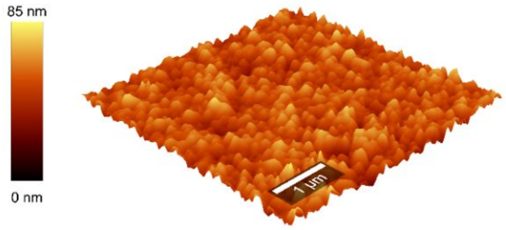

RMS=51 ± 8 nm
S 160 (int. no. 2024 23a)

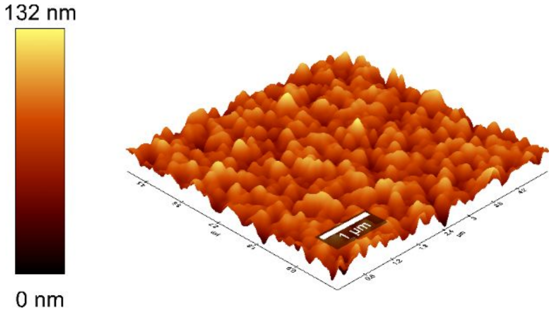

RMS=72 ± 17 nm
M 160 (int. no. 2025 I3)

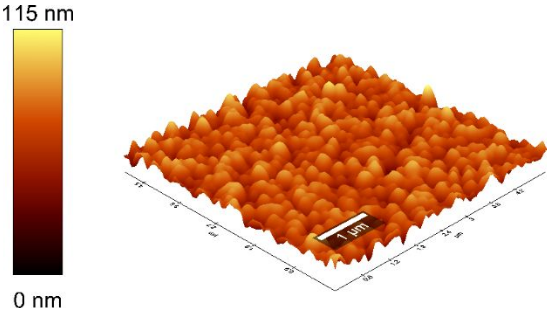

RMS=67 ± 13 nm
L 160 (int. no. 2025 C3)

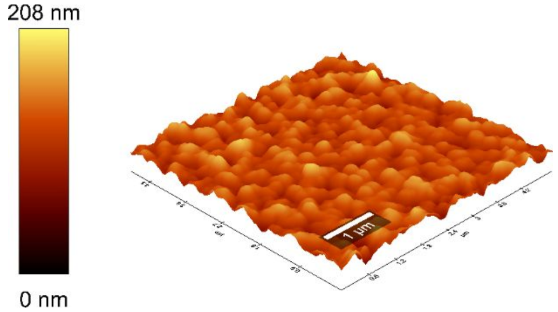

RMS=125 ± 19 nm
S 250 (int. no. 2025 G3)

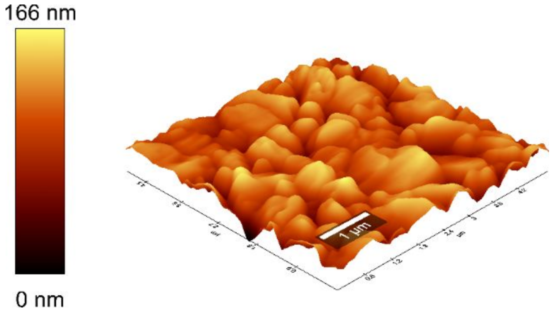

RMS=105 ± 23 nm
M 250 (int. no. 2025 B2)

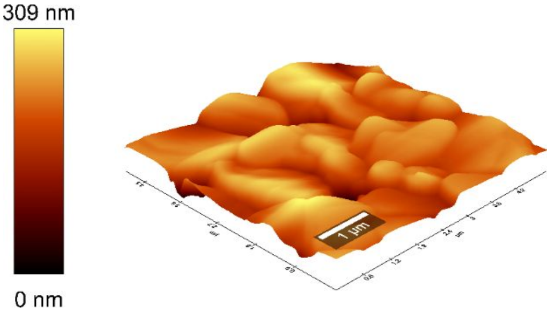

RMS=210 ± 40 nm
L 250 (int. no. 2025 A2)

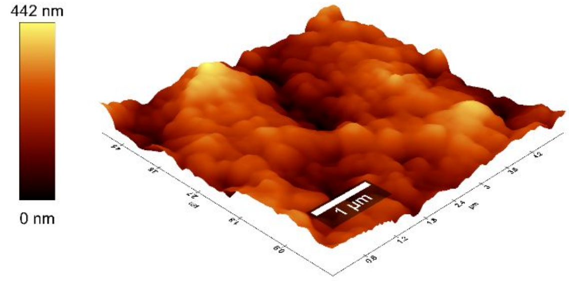

RMS=210 ± 70 nm
S 500 (int. no. 2024 C2)

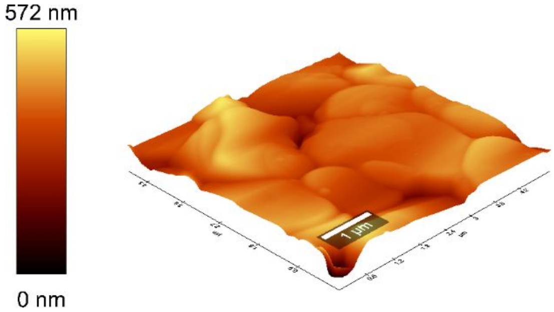

RMS=390 ± 60 nm
M 500 (int. no. 2025 D15)

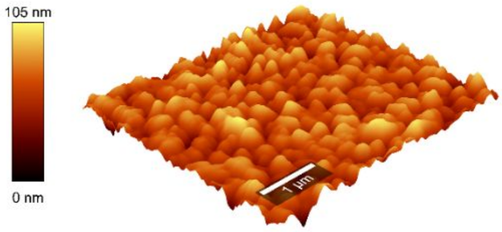

RMS=63 ± 14 nm
L 500 (int. no. 2024 E1)

Figure S9: Morphology of native roughness of MAPI samples.

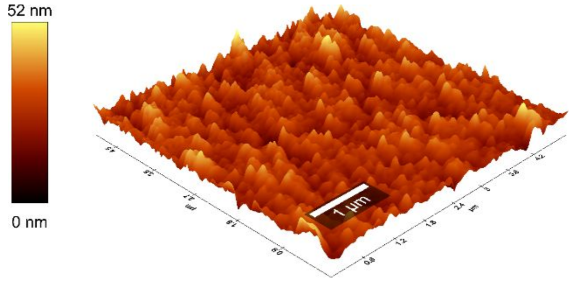

ZnO A, RMS = 26 ± 6 nm

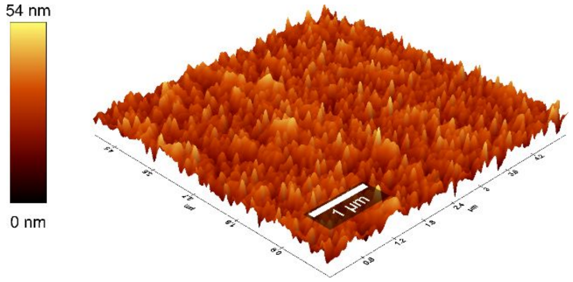

ZnO A with Au, RMS = 26 ± 6 nm

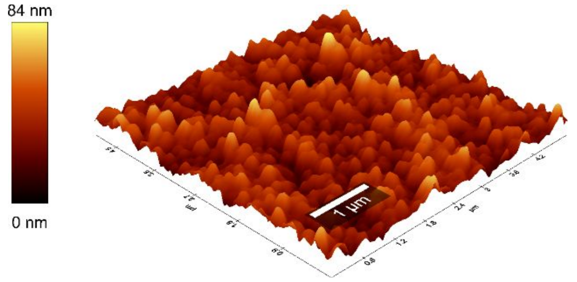

ZnO B, RMS= 34 ± 10 nm

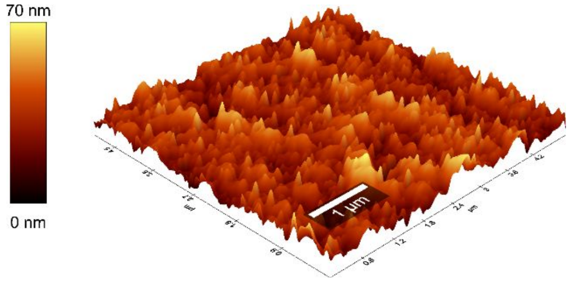

ZnO B with Au, RMS= 32 ± 9 nm

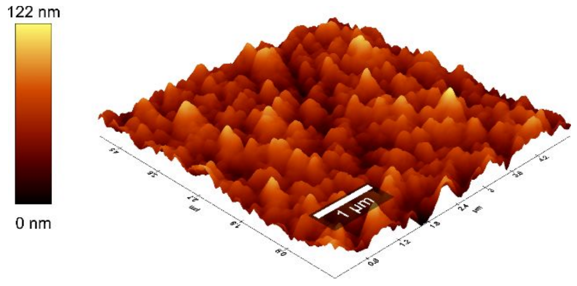

ZnO C, RMS = 50 ± 17 nm

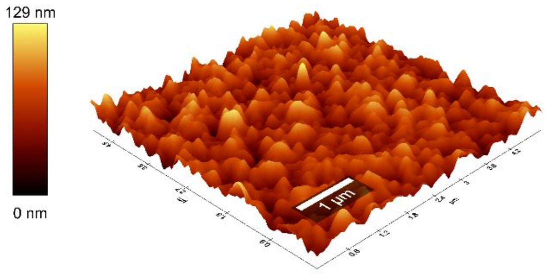

ZnO C with Au, RMS = 61 ± 17 nm

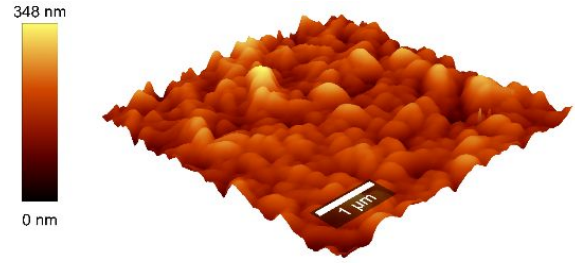

FTO, RMS = 173 ± 42 nm

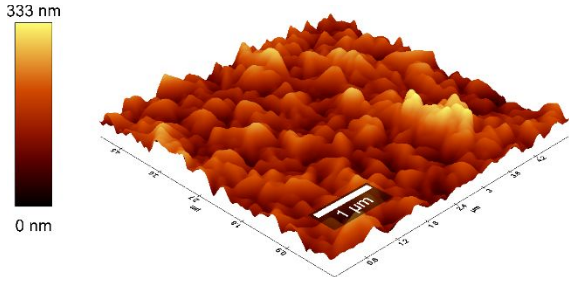

FTO with Au, RMS = 150 ± 50 nm

Figure S10: Morphology of nano-rough TCO substrates before (left image) and after (right image) the deposition of gold layer.

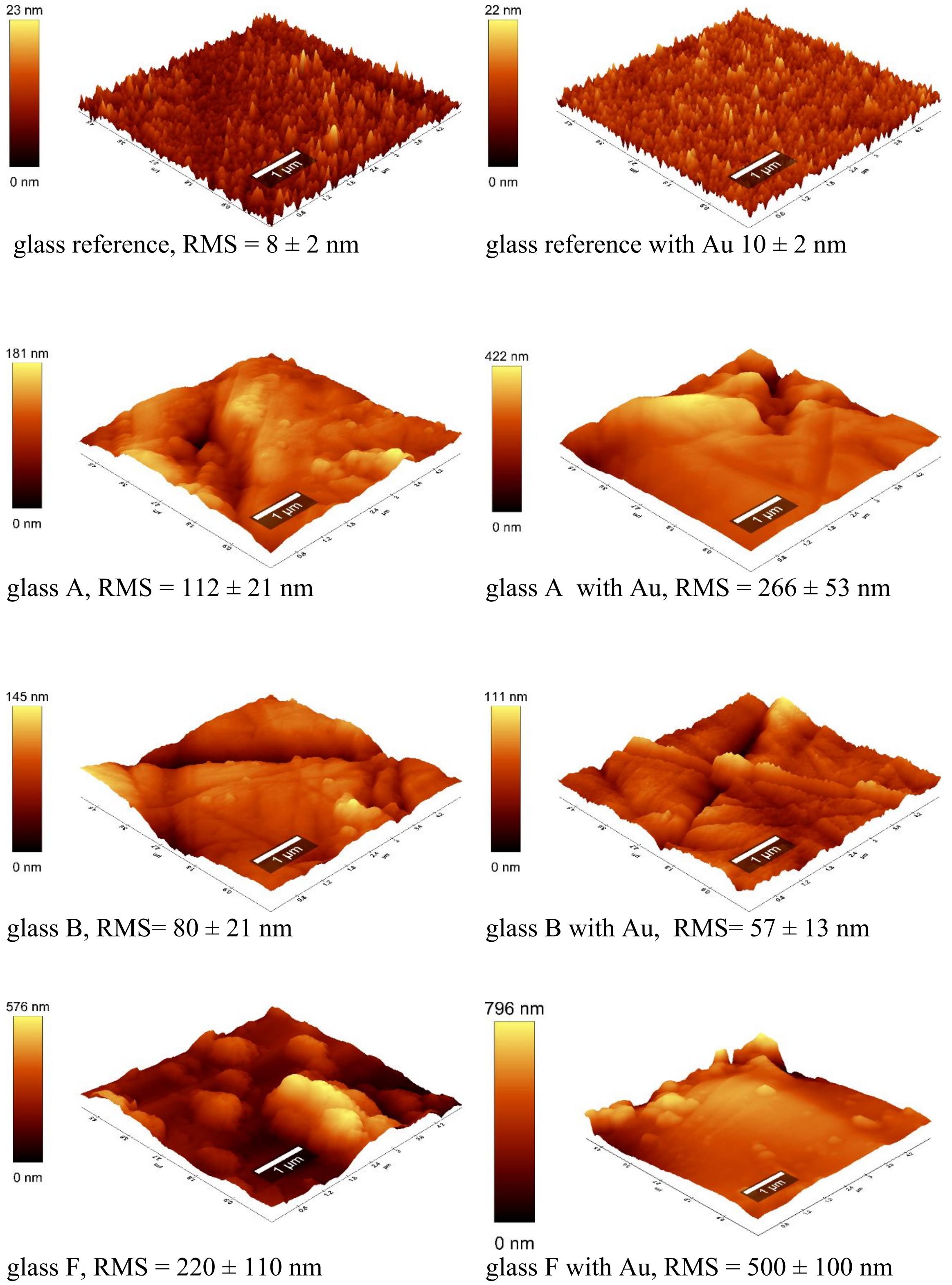

Figure S11: Morphology of micro-rough glass substrates before (left image) and after (right image) the deposition of gold layer.

Angular Distribution Function measurements

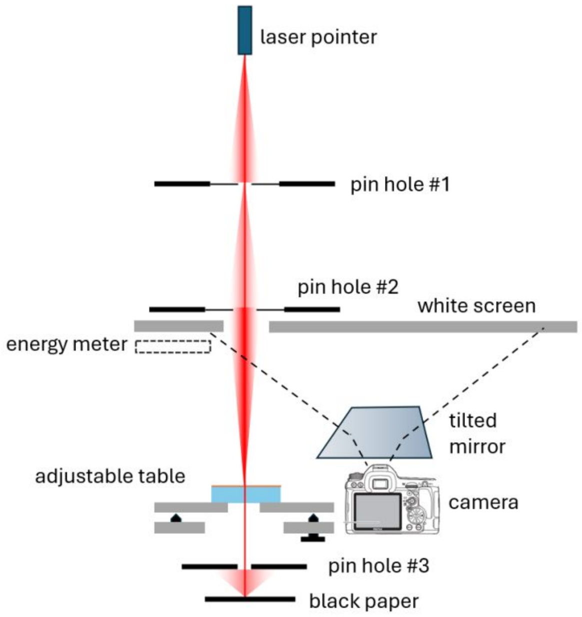

Figure S12: Angular distribution function measurement setup.

The measurement of Angular distribution function (ADF) consisted of a setup at Figure S12. Pinhole #1 was used for removing the scattered part of the laser, pinhole #2 was used for removing the diffraction pattern from pinhole #1. Adjustable table was used to direct the specular part of the reflection back into the pinhole, so that there was only the scattered part of the reflectance on the screen. Note that if the specularly reflected beam is falling on the screen, it will produce strongly disturbing halo effect on the resulting picture. This absolutely has to be prevented. For the purposes of measurement of the laser energy, the adjustable table was readjusted to direct the specular part into energy meter. Pinhole #3 plus black paper was there to trap all the transmitted beam. Distance between the sample and the screen was 10 cm. DSLR camera with CMOS chip was used to take pictures at ISO 800 and different exposure times ranging from around 0.1 s to 1 s. Pictures were stored in raw DNG format. Picture intensity along a straight radial line was first extracted by ImageJ software and then corrected for exposure time and for saturation effect by following equation (L_{true} is the corrected intensity in relative numbers, L_{exp} is the extracted value, and $exposure\ time$ is the time of the camera shutter):

$$L_{true} = \frac{-\ln(1-L_{true}/75)}{exposure\ time} \qquad (39)$$

The resulting set of curves for different exposure times were fitted in the region of their common overlap by polynomials and recalculated from pixel positions into angles by arctg function.

References

(1) Poruba, A.; Fejfar, A.; Remeš, Z.; Špringer, J.; Vaněček, M.; Kočka, J.; Meier, J.; Torres, P.; Shah, A. Optical Absorption and Light Scattering in Microcrystalline Silicon Thin Films and Solar Cells. *Journal of Applied Physics* **2000**, *88*, 148. https://doi.org/10.1063/1.373635.

(2) Beckmann, P.; Spizzichino, A. *Scattering of Electromagnetic Waves from Rough Surfaces*; Pergamon: London, 1963.

(3) Ritter, D.; Weiser, K. Suppression of Interference Fringes in Absorption Measurements on Thin Films. *Optics Communications* **1986**, *57* (5), 336–338. https://doi.org/10.1016/0030-4018(86)90270-1.
